# Novel Antigenic Targets of HPV Therapeutic Vaccines

**DOI:** 10.3390/vaccines9111262

**Published:** 2021-11-01

**Authors:** Ditte Rahbæk Boilesen, Karen Nørgaard Nielsen, Peter Johannes Holst

**Affiliations:** 1 Centre for Medical Parasitology, Institute for Immunology, University of Copenhagen, 2200 Copenhagen, Denmark; drb@sund.ku.dk; 2 InProTher APS, 2200 Copenhagen, Denmark; knn@inprother.com

**Keywords:** human papillomavirus, therapeutic vaccines

## Abstract

Human papillomavirus (HPV) infection is the cause of the majority of cervical cancers and head and neck cancers worldwide. Although prophylactic vaccines and cervical cancer screening programs have shown efficacy in preventing HPV-associated cervical cancer, cervical cancer is still a major cause of morbidity and mortality, especially in third world countries. Furthermore, head and neck cancer cases caused by HPV infection and associated mortality are increasing. The need for better therapy is clear, and therapeutic vaccination generating cytotoxic T cells against HPV proteins is a promising strategy. This review covers the current scene of HPV therapeutic vaccines in clinical development and discusses relevant considerations for the design of future HPV therapeutic vaccines and clinical trials, such as HPV protein expression patterns, immunogenicity, and exhaustion in relation to the different stages and types of HPV-associated lesions and cancers. Ultimately, while the majority of the HPV therapeutic vaccines currently in clinical testing target the two HPV oncoproteins E6 and E7, we suggest that there is a need to include more HPV antigens in future HPV therapeutic vaccines to increase efficacy and find that especially E1 and E2 could be promising novel targets.

## 1. Introduction

Human papilloma virus (HPV) infection is the known cause of the majority of cervical cancer cases, and is responsible for a growing number of head and neck cancers (head and neck squamous cell carcinoma, HNSCC, mostly oropharyngeal squamous cell carcinoma, OPSCC) [1,2], as well as penile, anal and vulvar cancers. In spite of the highly efficacious prophylactic HPV vaccine, HPV infection was still responsible for 690,000 new cancer diagnoses in 2018 [3].

HPV-associated cervical cancer accounted for over 300,000 deaths in 2018 [4], making it the fourth most common cause of cancer mortality in women worldwide and the most lethal HPV-associated cancer. Approximately 80% of these deaths occur in low and middle income countries, and the vast majority of the 42 countries where cervical cancer is the most common cause of cancer mortality are located in Sub-Saharan Africa and South-East Asia [4].

The current strategies to combat HPV infection are founded on prophylactic vaccination and routine screening for cervical HPV infection, neoplasia, and cancer. The available HPV vaccines are highly effective at preventing new HPV infections, but have no proven therapeutic effect [5]. This stipulates a problem for the millions of people already carrying a chronic HPV infection. Furthermore, the world-wide vaccine uptake is limited, with only few countries reaching vaccination coverage of 90% of the adolescent female population, which is the 2030 goal of the WHO Cervical Cancer Elimination strategy [6].

Routine screening is an efficient method for preventing HPV related cervical cancer by detecting and treating early cervical lesions. A screening goal is also included in the WHO Cervical Cancer Elimination strategy, with the aim that 70% of all women world-wide should receive twice-lifetime cervical screening by 2030 [7]. In a Danish context, for example, routine screening was shown to reduce the incidence of cervical cancer with about 70% [8,9] with a similar reduction in risk of death from cervical cancer for women who undergo screening, compared to those who do not [8,10]. However, routine screening for cervical neoplasia poses a logistical challenge for the health systems of many low- and middle-income countries, which also struggle the most with low vaccine uptake due to logistics as well as a high unit-price for the prophylactic HPV vaccines.

The current treatment options for cervical dysplasia is conization, follow-up by colposcopy and intensified screening, but the physical removal of the lesion is not a permanent method to eliminate the basal HPV infection, illustrated by frequent recurrence of lesions in women treated with conization [11]. Early-stage cervical cancers are commonly treated with surgery or radiation with or without low-dose chemotherapy [12], which cures approximately 80% of the patients (5-year disease-free survival) [13].

While the toolbox for managing HPV+ cervical cancer has been expanded and proven efficacious, HNSCC has only recently been linked to HPV infection, and has therefore historically received much less attention. There has been an overall decrease in HNSCC in the past decades, but the incidence of HPV+ HNSCC cases have markedly increased, in the US by 225% from 1988 to 2004 [1], and HPV is now detected in 55–75% of HNSCC [14,15]. Given this growth rate, it is not unlikely that HNSCC may surpass the annual number of cervical cancer in many high income regions in the near future, as it has already done in the US [16].

In contrast to cervical cancer, there are no established screening procedures to detect pre-HNSCC lesions. Oral HPV DNA has been suggested as a biomarker, but recent studies in cancer patients show a fairly low sensitivity of oral rinses (43–51%) [17], and studies on the correlation between oral HPV prevalence and risk of HNSCC are lacking [16]. Presence of serum antibodies against the non-structural HPV protein E6 have shown more promise as a biomarker for OPSCC, with a sensitivity of 88% [17]. Another possible biomarker the measurement of circulating HPV DNA in plasma which has been shown to correlate with later recurrence in patients cured for their primary OPSCC [18]. However, this approach has, to the best of our knowledge, not yet been assessed as a biomarker for unsuspected HNSCC.

A treatment for localized or locoregional HNSCC is chemoradiotherapy with high radiation intensity. While this therapy has a relatively high rate of curative success, especially for HPV+ cancers [1,19,20], the morbidity related to the treatment is substantial. Furthermore, for the approximately 20% of HPV+ HNSCC patients for whom chemoradiotherapy fails to cure the primary tumor, or who experience loco-regional relapse or distant metastases [21], the subsequent treatment options are rather limited. Checkpoint inhibitors (CPI) is standard treatment in this population, but with only sporadic curative effect and limited prolongation of survival [22,23], although the effect seem better in HPV+ than in HPV- HNSCC [24]. A similar poor prognosis is seen for incurable, metastatic or recurrent cervical cancers, where 5-year survival rates are between 5% and 15% [25], and CPI has shown limited effect, exemplified by an overall response rate of αPD-L1 treatment in patients with advanced cervical cancer of just 14.3% [26]. The use of CPI in combination with therapeutic vaccine strategies holds much promise and is reviewed later is this work (Section 6).

Despite effective prophylactic strategies, there is a major need for new and improved treatment options for HPV-associated disease. One of the promising strategies is therapeutic vaccination, capable of inducing immune responses toward relevant HPV proteins with the aim of removing HPV infection by killing HPV+ cells in a lesion or tumor. In a murine model, Spanos et al. has shown that one reason why chemoradiotherapy is more successful in HPV+ rather than HPV− HNSCC, is that it helps induce an immune response against the HPV+ tumor [27]. This supports the concept of induction of anti-tumor immune responses in HPV+ cancer patients. Additionally, it was recently shown that decreased local immune responses against HPV was associated with resistance to chemoradiotherapy and higher mortality in cervical cancer [28]. Altogether, this highlights the rationale of enhancing the HPV−specific immune responses to improve clinical outcomes, i.e., by therapeutic vaccination.

This review aims to provide an assessment of HPV−associated cancer immunobiology, HPV expression patterns in these cancers and the correlations of these factors with clinical outcomes. We will relate this to the implications of the choice of antigenic targets for HPV therapeutic vaccines, as well as to highlight relevant considerations and recent advances in novel antigens for HPV therapeutic vaccine design against both HPV infection and HPV-associated cancer. The delivery platforms of HPV antigen in a therapeutic vaccine context is diverse and highly important but is not the focus of this review. The readers are kindly referred to other publications and reviews for an overview of the various technologies applied in therapeutic HPV treatment research [29,30].

## 2. Expression and Immunogenicity of HPV Antigens in Patients Suggest Multiple New Antigenic Targets for Therapeutic Vaccines

Selecting the right antigens is an important decision, impacting the potential success of a therapeutic vaccine against HPV. Upon infection of the basal layer epithelial cells, HPV initially expresses the non-structural proteins E1 and E2. As infection progresses, and the cells move through the epithelial layer away from the basement membrane, E4 and E5 are expressed, followed by the oncogenes E6 and E7. Lastly, the capsid proteins L1 and L2 are expressed. The current prophylactic vaccines target L1, and some novel prophylactic vaccines in development target the more conserved L2 [31]. As HPV-associated neoplastic changes only occur following expression of high levels of E6 and E7, the vast majority of HPV−targeted immunotherapy has concentrated on these two antigens. E6 and E7 are known to be responsible for the malignant progression leading to HPV-associated cancer; E6 by binding and inhibiting the tumor suppressor p53 and E7 by inhibiting pRb and thereby allowing uncontrolled progression of cell cycle into S phase [32]. In the case of HPV+ OPSCC, elevated E6 expression correlated with shorter recurrence-free survival and higher risk of death [33] making the HPV oncogenes obvious targets for HPV+ cancer immunotherapy. However, as described later in this review, E6 and E7 targeted immunotherapy has shown limited success against HPV+ cancers. Further, E6 and E7 are only modestly expressed in the primary infected epithelial cells lining the basement membrane as compared to later in epithelial differentiation immediately preceding viral genome amplification. The persistent viral infection has succeeded in evading immunity to highly overexpressed E6 and E7 before it may be upregulated in malignant cells. Accordingly, targeting of these HPV proteins may therefore not be likely to clear the infection that caused the cancer in the first place. Hence, a broader antigen targeting strategy may be necessary to treat HPV+ cancers and to clear persistent pre-malignant infections.

E2 is expressed early upon infection and is important for regulation of transcription but is also known to suppress expression of E6 and E7 [34]. HPV DNA integration into the host genome leads to a disruption of the E2 open reading frame [35] resulting in a loss of E2 expression which consequently enables high level expression of E6 and E7 also in stem cells at the basal membrane [36]. Although integration is classically seen in cancer and advanced neoplasia (i.e., CIN 3, cervical intraepithelial neoplasia, stage 3, a classification of cervical lesions based on histopathology [37]), it has been reported in all stages of cervical lesions with frequencies increasing with increased severity [38,39]. Integration also varies between HPV types, where integration is more commonly seen for HPV18 than for HPV16 [40].

Integration with loss of E2 or other means of diminished E2 expression has been presumed necessary for oncogenic transition [41]. Consequently, E2 has been deemed irrelevant for therapeutic vaccines against HPV related cancers. E2 may therefore not be the ideal target for cervical cancers, but the expression pattern actually suggests that it would be highly beneficial to include E2 in a therapeutic vaccine given to patients with pre-cancerous HPV+ cervical lesions where E2 is typically intact. While integration and loss of E2 has been presumed to be prevalent in cervical cancers, around two-thirds of all HNSCC remain E2 positive [40,42], although the rates are highly various between studies, site of cancer and integration-detection-methods. Furthermore, the literature, although there are large variations between studies, suggests that far from all cervical cancers have disrupted E2 [38,40,43], reinstating the potential of including E2 in cancer vaccine designs. Intact E2 has been correlated with better survival and lower risk of recurrence in HNSCC, and intact E2 is more often seen for OPSCC than for other non-oropharyngeal HNSCCs [44].

**Figure 1:**
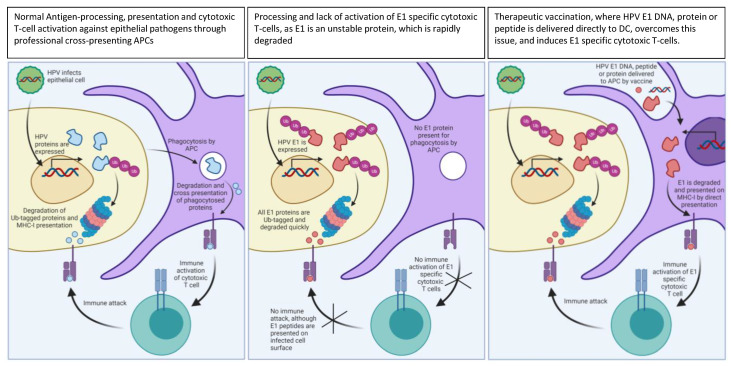
Normal processing and immune activation of viral or tumor antigens (**left** panel), of an unstable protein such as E1 (**middle** panel) and how therapeutic vaccines against such unstable proteins can induce beneficial cytotoxic immune responses (**right** panel). Figure created with Biorender.com, accessed on 1 November 2021.

E1 is crucial for viral replication and is continuously expressed in the HPV infected basal epithelial cells, considered stem cells [45]. Recently, it has been suggested that E1 plays a role in carcinogenesis [46]. E1 is the largest of the HPV proteins (649 amino acids), making the number of potential T cell epitopes across HLA alleles much larger compared to the smaller E6 and E7 proteins (154 and 98 amino acids, respectively). Hence, the size and continuous expression of E1 would make it an attractive target for therapeutic HPV vaccination. However, E1 has suffered the same fate as E2 and been surpassed by E6 and E7 as the main antigens of interest. One reason may be that E1 protein expression has not been reported in cervical HPV+ lesions. We have been unable to find studies reporting detection of E1 protein expression, but one study shows a lack of E1 specific antibody responses in patients with early HPV+ lesion and cervical cancer, suggesting low or minimal past presence of E1 protein [47]. This may be consistent with the immunobiology of papilloma virus infection, as studies on bovine papilloma virus show that E1 is an unstable protein that is ubiquitinylated and rapidly degraded in papillomavirus infected cells [48,49]. It is still unverified that the same occurs in HPV infection, but as depicted in Figure 1, rapid degradation of E1 could explain why no antibody responses are raised against E1, despite E1 expression being needed for HPV replication. Crucially important, rapid degradation of E1 does not mean that E1 is a poor target for cytotoxic T cell mediated immune responses. Degradation of protein through the proteasome is a prerequisite for epitope-presentation on MHC-I on the surface of infected cells. This is exemplified by the fact that targeting of proteins for proteasomal degradation by ubiquitinylation has been used as an adjuvant technology in a therapeutic vaccine against the cotton-tail rabbit papillomavirus (CRPV) E6 [50]. The T cell adjuvant Invariant Chain was also shown to increase CD8 T cell responses by enhancing the proteasomal degradation of vaccine antigens through increased ubiquitinylation [51]. In agreement with the suggestion that E1 may be an interesting T cell target, Ma et al. recently showed that E1 specific T cells could be detected in 59% in a cohort of cervical cancer patients, and importantly, these responses correlated with improved progression free survival and overall survival [52].

While it is reasonable to use expression patterns as a starting-point for selection of relevant target antigens, expression alone may be insufficient for determining optimal antigens. Another approach to antigen identification has been to look at the specificities of the T cell responses induced by natural HPV infection, and how these responses correlate with clinical outcome. The vast majority of past studies on immune responses in HPV patients have focused on E6 and E7 responses in cervical cancer patients. Nakagawa et al. reported that cytotoxic T cells targeting E6 have shown to correlate with clearance of chronic HPV infection [53] and T cells specific to E6 and E7, especially if infiltrating the tumor, have shown to correlate with reduced risk of metastasis [54]. Lately, more attention has been given to the increasing number of OPSCC patients, where CD8 T cell responses against E7 have been correlated with longer disease-free survival [55].

However, more recent studies have investigated immune responses of other specificities than E6 and E7. In a study of patients with low-grade squamous intraepithelial lesion (LSIL; a classification of cervical lesions based on cytology [37]), Woo et al. showed that E6 responses correlated with persistence of lesion (in seeming contrast to Nakagawa et al. cited above), while E2 specific T cell responses were associated with lack of progression into high-grade squamous intraepithelial lesion (HSIL) [56]. Similarly, Dillon et al. showed that patients who had recently resolved their cervical dysplasia had higher E2 specific T cell responses than patients with persistent or progressive disease [5], supporting the role for E2 in immunotherapy of pre-cancerous cervical lesions. The conflicting reports linking E6 responses to progression and clearance respectively in European and Asian populations underscores that population genetics at the HLA level could be more important than the cellular function of the protein with respect to immune recognition and association with prognosis.

In contrast to cervical disease, E1 antibody responses have been detected in HPV+ OPSCC patients [57], suggesting that there may be other fundamental differences in HPV behavior in different infection sites than just integration and subsequent loss of E2. In general, the profile of immune response specificities seems broader for OPSCC. Andersson et al. repeatedly found E1, E2 and E4–7 antibody responses in OPSCC patients [57,58,59], and the presence of antibodies against E1, E2 and E6 correlated with better clinical outcomes [59]. Bhatt et al. detected CD4 and CD8 T cell responses against E1, E2, E4, E5, E6, E7 and L1 proteins in OPSCC patients, in a hierarchy favoring E1 and E2 responses over E6 and E7, especially after chemoradiotherapy [60]. Keeping in mind that HPV presence enhances the sensitivity of chemoradiotherapy due to increased T cell immune responses against HPV antigens (only E6/E7 investigated) [27], it is likely that E1 and E2 responses also have a role to play in the clearance of HPV+ OPSCC or that such responses predict the presence of intact HPV genomes that in turn predicts response to therapy.

E4 and E5 have historically not been given much attention, and given their size (smaller than E6 and E7), they are less likely to contain more potential epitopes than E1 and E2. E4 expression has been detected in the middle and upper parts of HPV infected epithelia and is not expressed during early HPV infection or during cancer. The function of E4 is not clear, but it is suggested to have cause disruption of the keratin network and thereby play a role in viral release, and to be involved in cell cycle arrest [61]. E5 function is also poorly understood, but it has been shown to down-regulate surface MHC-I [62] and could thereby help to mediate the immune evasion of HPV infection and HPV+ cancer, making it a putative oncogene. The expression patterns of E5 are equally unclear, but it is sometimes lost during integration together with E2 [63] and in contrast to the two other oncogenes, E6 and E7, E5 is far from always expressed in HPV+ cancers (cervical lesions [64] and HNSCC [65]).

A study on a small population of women with cervical dysplasia showed that, while there were detectable CD8 T cell responses toward E6 and E7, none of the women had responses toward E4 [66]. Bhatt et al. also found E4 to be the least immunogenic HPV antigen in their cohort of OPSCC patients, while E5 T cell responses, especially CD8, were more frequent [60]. In terms of relevance for patient outcomes, we have been unable to find studies correlating E4 expression or cellular immune responses to clinical outcome.

A study of patients with HPV+ tonsillar cancer showed E5 expression in a fairly large proportion of patients, but no correlation between E5 expression and clinical outcome [67]. On the contrary, a study in OPSCC patients showed correlation between E5 expression and recurrence-free survival [65], but this correlation could potentially also be due to a higher level multicopy episomal HPV vs. integrated HPV DNA (where E5 is often disrupted), as is often seen for OPSCC, causing a higher level of general HPV antigen expression and thereby better clinical outcome. Additionally, a recently published study found that tumors from HPV+ HNSCC patients contained specific CD8 T cells specific for E5, as well as E2 and E6, and the authors argue that other HPV antigens than just E6 and E7 should be included as vaccine antigens to maximize the breadth and thereby the efficacy of therapeutic vaccines [68]. Studies correlating E5-directed CD8 T cell responses and clinical outcome could be highly informative.

## 3. Challenges and Opportunities of HPV Therapeutic Vaccines

Until recently, there has been no standard monitoring or detection of non-malignant HPV infections, and therefore little incentive to develop therapies against pre-CIN HPV infection. However, studies have shown that HPV status is a better predictor than pap stains for risk of cervical cancer [69,70], causing a shift in the guidelines for cervical cancer screening programs toward HPV detection [71,72]. The Netherlands, Australia [73], Norway [74] and New Zealand [75] have implemented this in the national cervical cancer screening, or are in the process of doing so, and health authorities in the US [72], UK [76], Sweden [77] and Denmark [78] have recommended to follow suit.

The current guidelines for management of HPV− positive low-grade abnormalities remain conization and increased monitoring [71], but the detection of more HPV infections creates an opportunity for a more targeted HPV− specific therapeutic approach as an alternative to just waiting for higher-grade abnormalities to occur and trigger subsequent invasive treatment. Furthermore, conization and monitoring does not clear the HPV infection and does therefore not remove the risk of recurrence of abnormalities [11]. A therapeutic vaccine effectively removing any HPV infection would be highly beneficial in the context of modern screening programs.

A challenge for vaccines targeting persistent HPV infection and pre-cancerous stages of CIN is the availability of animal models to study vaccine efficacy. The most common animal model for therapeutic effect is the TC-1 tumor cell line in C57BL/6 mice, which expresses HPV16 E6 and E7. Another common, but less frequently used model, is the C3 tumor cell line, also allogenic to C57BL/6, which expresses the full HPV16 genome [79].

Besides being a tumor model and not a model for infection, the nature of an inbred mouse model also poses a translational barrier. The H-2Db restricted E7(49–57) epitope may account for most of the therapeutic effect seen in these models, especially in TC-1, which constitutes an obvious challenge in translating therapeutic effect from mouse studies into an HLA-diverse human population. An example of another recently developed model, is the mouse tonsil-derived epithelial expressing HPV16 E6 and E7 genes [80]. Although this model could have advantages in terms of better translation toward human HNSCC, it still suffers from the same issues of being specific for the inbred C57BL/6 strain, and of not being suitable for studies on pre-malignant chronic infections. Another potential animal model employs cotton-tail rabbit PV. Here, it was found that any of the E1, E2, E6 or E7 encoding DNA vaccines could elicit therapeutic efficacy [50]. An alternative to mouse or rabbit models is the Macaca fascicularis monkey model of prevalent or persistent genital infection, but it is likely that better animal models would increase our ability to evaluate the therapeutic potential of vaccine candidates prior to clinical trials [81].

## 4. Clinical Attempts at Treating Early Cervical Dysplasia (CIN1–3) by Targeting E6 and/or E7

Although the translation of therapeutic HPV vaccines from animals to humans is challenging, increased numbers of candidates have been tested in clinical trials over the past years. Table 1 provides an overview of the clinical responses achieved by the more promising therapeutic HPV vaccines tested in the clinic over the past years.

ISA101 is based on long synthetic peptides covering the entire HPV16 E6 and E7. The vaccine produced rather promising results in a phase I/II study on patients with high-grade vulvar or vaginal intraepithelial neoplastic lesions with or without co-treatment with the immune modulator imiquimod, commonly used for genital warts (imiquimod did not affect the outcome of ISA101 treatment). Here, vaccine-induced clinical responses were observed in 53–60% of patients at 3 months and in 52–79% patients at 12 months, of whom 53–60% displayed a complete histologic response at 12 months after the last vaccination [82,83]. Importantly, the magnitude of vaccine-induced T cell responses correlated with complete regression, and viral clearance occurred in all but one of the patients with complete histological clearance. ISA101 has, to the best of our knowledge, not been tested in a placebo-controlled clinical trial.

Another protein-based vaccine, GTL001, encodes HPV16 and 18 E7 proteins fused to inactive Bordetella pertussis adenylate cyclase as vaccine vector, to enable direct targeting of CD11b+ cells (dendritic cells), has previously shown clearance of HPV16+ tumors in mice [84]. A placebo-controlled phase II trial in women with HPV16 and/or 18 infection and normal cytology or LSIL, showed a reduction of viral load with GTL001 in combination with imiquimod [85], but unfortunately, no clinical difference was observed between therapy and placebo groups [86]. This study emphasizes the importance of placebo control when investigating therapeutic vaccines targeting pre-malignant HPV infections and CIN, as these infections and neoplasia are likely to spontaneously resolve.

A range of DNA–vectored HPV therapeutic vaccines have also been tested against CIN2/3. Most often these encode E6 and E7 from HPV16 and 18, either alone as for VGX-3100, or in sequence with genetic or molecular adjuvants. VB10-16 encodes the human chemokine MIP-1alpha, which enables direct targeting of APCs, and GX-188 encodes the Fms-like tyrosine kinase-3 ligand together with the HPV antigens. VGX-3100 showed histological regression in 49.5% (53/107) of treated CIN 2/3 patients compared to 30.6% (11/36) in the placebo group at week 36 in a phase IIb trial [87]. In total, 91% of the women who had experienced regression and avoided excision had no detectable HPV DNA or HSIL recurrence after 18 months [88]. GX-188 showed histological regression in 67% (35/52) of CIN3 patients after 18 months, of which 77% (27/35) cleared their HPV infection (phase II) [89]. Similarly, 8 out of 14 patients treated with VB10-16 showed regression to CIN1/0, while 12 out of 14 patients showed overall reduction in lesion size 12 months after treatment initiation, and encouragingly, HPV16-specific T cell responses correlated with lesion regression in a phase I/II study [90,91]. However, neither of these two therapeutic vaccine candidates have been evaluated against placebo yet.

In the field of viral vectored therapeutic vaccines, we find TG4001, an MVA encoding HPV16 E6 and E7 together with IL-2, and TA-HPV, a vaccinia encoding HPV16 and 18 E6 and E7 which is used as a booster after HPV16 E7 DNA prime. TG4001 was tested in a placebo-controlled phase II trial on patients with CIN2/3 and showed 18.2% complete histological clearance in HPV16+ mono-infected patients after 6 months, compared to only 4% in the placebo group. Furthermore, the TG4001 vaccine provided significantly higher rates of complete viral DNA clearance compared to placebo after 30 months (43% (55/127) vs. 32% (20/62)) [92]. Importantly, this study also showed that it is harder to achieve complete regression in CIN3 than CIN2 patients, supporting the strategy to treat HPV disease early. A small phase I study of the DNA_E7_+TA-HPV prime boost strategy gave complete histological regression in 5/12 HPV16+ CIN2/3 patients. This study also showed increased vaccine-specific immune responses and CD8 T cell infiltration in lesion, but no clear correlation between immune responses and lesion regression [93]. Overall, the sum of studies suggests the potential of eliciting a therapeutic effect, but consistent efficacy is still imminent.

**Table 1:** Summary of recent clinical trials of HPV therapeutic vaccines against cervical dysplasia (CIN1–3).

Vaccine	Antigen(s)	Delivery Method and Adjuvants	Co-Treatment	Patient Population	Clinical Phase	Conclusion(s)	Trial ID	References
VB10.16	Full length HPV16 E6 and E7 coupled to MIP-1α	DNA Encoding HPV antigen linked to human chemokine MIP-1alpha-targets APCs directly		CIN2/3	I/II	In total, 12 out of 14 patients showed a reduction in the lesion size 12 months after treatment initiation. Histopathological regression to low grade neoplasia (CIN1) or no disease was seen in 8 patients. Of the 6 patients that has not regressed to CIN1 or less at 12 months, 5 patients showed upregulation of PD-L1 in the lesions, and three of these patients had also persistent co-infection with other high-risk HPV strains. 16/17 patients had increased HPV16 T cell responses post vaccination.	NCT02529930	[90,91]
GX-188E	HPV16 and 18 E6 and E7	DNA Encoding HPV antigens and Fms-like tyrosine kinase-3 ligand		CIN3	II	Histologic regression in 67% of patients. 73% of patients with regression showed HPV clearance.	NCT02139267	[89]
VGX-3100	HPV16 and 18 E6 and E7	DNA		CIN2/3 (placebo controlled)	IIb	Histological regression in 49.5% of treated vs 30.6% of placebo after 36 weeks. 91% of the women who had experienced regression and avoided excision had no detectable HPV DNA or HSIL recurrence after 18 months.	NCT01304524/EudraCT 2012-001334-33	[87,88]
TA-HPV + DNAE7	HPV16 and 18 E6 and E7	HPV16 E7 (DNAE7) at study weeks 0 and 4, followed by a recombinant vaccinia boost expressing HPV16 and HPV18 E6 and E7 (rVacE6E7; TA-HPV) at study week 8		HPV16+ CIN2/3	I	In total, 7/12 patients generated vaccine specific immune responses, and 5/12 patients showed complete histological regression-however no correlation between responses and regression reported. Increased CD8 T cell infiltration in lesion after vaccination.	NCT00788164	[93]
ISA101	synthetic long peptides covering the entire HPV16 E6 and E7	Peptide Freunds incomplete adjuvant	+/- 5% imiquimod cream	High-grade vulvar or vaginal intraepithelial neoplastic lesions	I/II	Vaccine-induced clinical responses were observed in 53–60% of patients at 3 months and in 52–79% of patients, of whom 53–60% displayed a complete histologic response at 12 months after the last vaccination. Vaccine-induced T cell responses were significantly stronger in patients with complete responses. Importantly, viral clearance occurred in all but one of the patients with complete histologic clearance.	NL21215.000.08	[82,83]
GTL001	HPV16 and 18 E7	Proteins fused to inactive Bordetella pertussis adenylate cyclase as vaccine vector (direct targeting of CD11b+ cells)	5% imiquimod cream	Women With Normal Cytology or ASCUS/LSIL. Aimed at clearance of HPV616/18 infections (placebo controlled)	II	No clinical difference observed between therapy and placebo group.	NCT02689726/EudraCT 2013-003358-25	[84,85,86]
TG4001	full length HPV16 E6 and E7	MVA Encoding HPV antigens and IL-2		CIN2/3 (placebo controlled), 13 different hrHPV types	II	Histologic complete resolution of CIN2/3 in 18% of HPV16+ patients after 6 months (4% for placebo). Viral clearance in 43% (55/127) of CIN2/3 patients after 30 months (32%, 20/62 for placebo group).	NCT01022346/EudraCT 2008–006946-24	[92]
VTP-200	conserved elements of E1, E2, E4, E5, E6 and E7 proteins representing HPV genotypes 16, 18, 31, 52 and 58	Encoded into ChAdOx1 and MVA		low grade cervical lesions (placebo controlled)	I/II	No results available yet.	NCT04607850	

## 5. Pre-Clinical and Clinical Attempts at Treating HPV Infection and Early Cervical Dysplasia by Targeting Other Antigens Than E6/E7

As outlined previously, E1 and E2 are expressed from early infection up until malignant transformation, making them relevant target-antigen candidates for pre-malignant therapy. More importantly, E1 and E2 are expressed in the primary infected cells at the basal membrane which are the stem cells maintaining the infection. Failure to remove infection from these cells may therefore result in an overall failure at removing the underlying infection that led to the neoplastic changes, thereby increasing the risk of recurrence of neoplastic changes.

An intriguing approach is a broadly reactive therapeutic vaccine, capable of targeting and clearing the infection from multiple different HPV types. This has been attempted by Hancock et al., by encoding conserved elements of E1, E2, E4, E5, E6 and E7 proteins representing HPV genotypes 16, 18, 31, 52 and 58 into a viral-vectored vaccine. The vaccine was immunogenic in mice, and CD8 T cell immune responses against the vaccine antigens were detectable in a number of patients with hrHPV infection. It will be interesting to see indications of the coverage and the therapeutic effect on early lesions in the current phase 1b/2 trial (under the name VTP-200) [94,95].

A previous study in our group used a different approach to achieve broad coverage by creating a common ancestor sequence of E1 and E2 covering a wide range of HPV genotypes. The therapeutic potential of the vaccine was tested in papillomavirus infected Macaca fascicularis monkeys, but the vaccine only succeeded in inducing multi-genotype protection in less than half of the animals. However, a few animals acquired CD8 T cells toward a specific genotype of papillomavirus infection and these were cured of their infection [96].

Although a therapy with broad coverage across HPV types has obvious advantages, it might be at a cost of cross-HLA coverage and efficacy against any specific type, as, everything else being equal, a type-specific vaccine would always be expected to outcompete cross-reactive vaccines for their designed target. In a follow-up study, our group systematically investigated vaccine induced CD8 T cell cross-reactivity in outbred CD1 mice and found that the vaccine-antigen and the HPV antigen could have up to 10% diversity to the immunogen without losing antigenicity, but cross-reactivity dropped dramatically when diversity increased further [97]. The relatively low sequence similarity between various hrHPV types poses a challenge for this strategy, as cross-reactive vaccines would need to bridge close to 30% sequence diversity. Therapeutic vaccines encoding fractions of multiple HPV genotypes should have an advantage of cross-type coverage but may not be efficacious in all patients. Furthermore, the inclusion of HPV− detection and HPV− typing in cervical cancer screening programs, will lead to more women getting diagnosed with an identified genotype of pre-CIN HPV infection. This stipulates a window of opportunity for type-specific therapeutic vaccines.

A principally promising example of such a vaccine is a modified vaccinia Ankara (MVA) vector encoding full length HPV16 E2. This was tested in a phase III clinical trial on women with CIN1-3. 89.3% showed complete elimination of CIN, and HPV DNA was undetectable in in 83% of patients [98]. However, the vaccine was administered locally (i.e., in uterus, urethra or anus) with weekly administration for 6 weeks. Accordingly, it is possible, that the effect was not due to vaccine-induced immune responses, but rather due to E2 mediated suppression of E6/E7 expression by the vaccine encoded E2 protein. As it is unclear what the long-term results for this patient cohort is, this unanswered mechanistic question might hold the answer to why this therapeutic vaccine strategy has not been taken further in clinical development since 2014.

To the best of our knowledge, no other therapeutic vaccines targeting antigens other than E6 and E7 have been published for therapeutic effect in a clinical setting.

## 6. Clinical Attempts at Treating HPV+ Cancers by Targeting E6 and/or E7

Traditionally, cervical cancer has been the main focus for research on HPV-associated disease and development of prophylaxis and therapy against such. However, as already outlined, HPV− associated HNSCC is on the rise and have received increasing interest as targets for HPV− directed anti-cancer therapy. Table 2 summarizes the most recent clinical trials using therapeutic vaccines to target HPV+ cancers.

ISA101, the synthetic long peptides of HPV16 E6 and E7 described above, was tested both in patients with cervical cancer and HNSCC as well as other HPV+ cancers. One phase I/II trial investigated the therapeutic effect of ISA101 in 77 patients with advanced, metastatic, or recurrent cervical cancer as an add-on to chemotherapy. The treatment resulted in regression in 43% and stable disease in 43% of the patients, and importantly, vaccine-specific T cell responses correlated with increased probability of survival. The patients with higher than median vaccine-induced immune responses had a median overall survival of 16.2 months, compared to 11.2 months for patients with lower than median responses [99]. In comparison, the overall survival for similar patients receiving chemotherapy alone is in other studies reported to be 10–12.9 months [100].

In many cases, patients with advanced, metastatic, or recurrent HPV+ HNSCC cancer are treated with immune checkpoint inhibition (CPI). As HPV+ cancers express non-self-antigens, as T cell responses against these have shown prognostic benefit, and as checkpoint molecules are expressed within cancers, the rationale for unleashing these responses by CPI treatment is clear [24], in particular in combination with a therapeutic vaccine further enhancing the HPV− specific responses [101]. ISA101 was tested in combination with αPD1 in a phase II trial in patients with incurable HPV16-positive cancer, mostly OPSCC with an overall response rate of 33% and a median overall survival of 17.5 months [102]. Response rates in similar patients are 16–22% with αPD1 treatment alone [22,103]. Notably, the response rate was higher in patients with PD-L1 positive than PD-L1 negative tumors. In contrast to what was seen for ISA101 with chemotherapy but not CPI described above, anti-vaccine T cell responses did not correlate with therapeutic efficacy, pointing toward tumor micro-environmental factors as being crucial for vaccine effect.

Another peptide-based vaccine in clinical testing is PDS0101 where HPV16 E6 and E7 peptides are delivered in liposomal nanoparticles. This vaccine is currently being tested in a phase II trial in a triad-combination with Bintrafusp alfa (a TGF-β and PD-L1 inhibitor) and IL-12 treatment on patients with advanced HPV− associated malignancies who have already failed chemotherapy. Preliminary results show an overall response rate of 55.6%, and tumor reduction in 66.7% of patients after a median of 8 months follow-up [104]. CPI naïve patients show a higher response rate, potentially because these patients have not already benefitted from the effect of CPI prior to the trial. However, among the 12 patients who had previously failed CPI treatment, the overall response rate was 42% compared to 5–12% at current standard of care for this patient population. Furthermore, 10 out of the 12 patients were still alive at 8 months, which is promising when keeping in mind that the median survival for this type of patients is typically 3–4 months [105]. It will be exciting to follow the future reports from this study, and the two other ongoing phase II studies on PDS0101 in combination with αPD-L1 against recurrent or metastatic HNSCC (NCT04260126) and with chemo-radiotherapy against advanced local cervical cancer (NCT04580771).

Two of the DNA-based vaccines tested in CIN patients, VB10-16 and GX-188, are also evaluated in patients with advanced HPV+ cervical cancer. While the VB10.16 phase I/II trial is currently ongoing with no reports on preliminary results [106], GX-188 together with αPD1 showed lesion regression in 42% of patients after 24 weeks, with partial response in seven and complete response in four of 26 patients [107].

An emerging strategy in the field of immunotherapy is personalized approaches by manipulation of immune cells from the individual patient. Such strategies have also found their way to HPV therapy, with SQZ-PBMC-HPV-101 in clinical testing. SQZ-PBMC-HPV-101 is based on their cell-squeeze technology where HPV16 E6 and E7 antigens are delivered to the cytosol of patient APCs ex vivo by temporarily disrupting the cell membrane by mechanical squeezing through a chip at high speed [108]. 12 patients with incurable HPV16+ cancer were treated with SQZ-PBMC-HPV-101 and 4 patients achieved stable disease [109].

TG4001, the MVA vector encoding HPV16 E6, E7 and human IL-2, is currently being tested in combination with αPD-L1 on patients with a range of recurrent or metastatic HPV+ cancers in a phase Ib/II trial where preliminary results report 23.5% clinical response rate after 12 weeks (1/34 had complete clinical response, 7/34 had partial response). More than half of the patients showed no disease progression at 12 weeks compared to an expected mean progression-free survival of 8 weeks in this population with current treatment. An important note from the preliminary study is that clinical responses correlates with CD3+ and CD8 cell infiltration into the tumor, stressing the unsurprising although important notion that the effect of therapeutic vaccines depend on the CD8 T cells infiltrating the lesion [110,111]. The use of therapy induced CD8+ T cell infiltration could be considered a useful early surrogate for efficacy non-placebo-controlled early clinical studies.

ADXS11-001 is a Listeria monocytogene bacteria engineered to secrete a truncated listeriolysin O fused to HPV16 E7, to target the HPV16 E7 for phagocytosis by APCs. ADXS11-001 has been tested in a range of phase I and II trials in various HPV+ patient populations, summarized in a mini-review by Galicia-Carmona et al. [112]. Most recently, a phase II study in patients with recurrent cervical cancer, who were already on chemo-radiotherapy, showed a 12-months overall survival of 30.9–38.9% and a mean progression-free survival of approximately 6 months independent of whether ADXS11-011 was given as monotherapy or co-administered with cisplatin [113]. A phase III trial of ADXS11-011 in patients with advanced cervical cancer is currently ongoing (NCT02853604) and results are expected in 2021.

Another viral-vectored vaccine that has recently been clinically evaluated is the HB-201/202 combination, where and HPV16 E6/E7 fusion protein is encoded into lymphocytic choriomeningitis virus (HB-201) or Pichinde virus (HB-202) enabling a heterologous arenavirus vectored prime-boost regimen. The phase I study investigated a single-dose (HB-201) or a prime-boost regimen in heavily pretreated HPV+ cancer patients. The majority of patients had OPSCC and an average of three prior lines of therapy, including CPI for most patients and the majority did not receive CPI during the study. When fully implemented the treatment generated an impressive level of circulating HPV16 E6/E7 specific CD8 T cells of up to 40% of circulating CD8 T cells in one case and in most cases around 1000 spots/million in ELISPOT assays, although in the early analysis there was a high number of patients not available for follow-up analysis. With such a promising immune response, the clinical outcomes were however sobering. The overall response rate in HNSCC patients was 18.2% and the mean progression free survival was 3.5 months, which does not differ much from the survival rates with current standard of care [114]. It remains to be understood how the regimen could expand large numbers of T cells that were fully functional directly ex vivo, yet only exerted moderate anti-tumor efficacy. Explanations for this underwhelming result could be that the tumors were relatively immunogenic themselves or that they were advanced. Another possibility could be that they were targeted after progression on CPI, as it has been shown that prior treatment with CPI in patients with a low or insufficient anti-tumor T cell response may hamper the efficacy of a subsequent therapeutic vaccination [115]. In this regard it will be interesting to see the HB-200 regimen applied to earlier disease states. Notably, the specificity and anti-tumor efficacy of the additional CD8 T cells induced are unknown and it should be considered that the amazing epitope breadth and response potency [116] which can be elicited by non-invasive strains of arenavirus, may elicit responses that have poor relevance for cancer control. The relevance of massively augmented T cell frequencies can also be questioned, as in pre-clinical models, the Lymphocytic Choriomeningitis Virus (LCMV) was no more effective than an adenoviral vaccine engineered for effective and broad epitope presentation in control LCMV epitope positive melanomas [117].

Although it is hard to compare the different clinical trials directly, due to differences in patient population, co-treatments, and follow-up times, it is evident that therapeutic vaccinations seem to prolong patient survival. There is an overall trend that it is possible to achieve sometimes profound responses in specific subsets of patients, but it appears that there is still some way to go in the quest for curative therapeutic vaccination treatment, especially for advanced HPV+ cancers. While some level of responses seem achievable in many regimens, the underlying differences between responders and non-responders are still unresolved. Considering the important role of host genetics at the HLA level on primary control of HPV infection, it is unfortunate that the therapeutic studies typically do not report HLA typing.

**Table 2:** Summary of recent clinical trials of HPV therapeutic vaccines against HPV+ cancer.

Vaccine	Antigen(s)	Delivery Method and Adjuvants	Co-Treatment	Patient Population	Clinical Phase	Conclusion(s)	Trial ID	References
VB10.16	Full length HPV16 E6 and E7 coupled to MIP-1α	DNA Encoding HPV antigen linked to human chemokine MIP-1alpha-targets APCs directly	aPDL-1	Advanced, non-resectable cervical cancer	I/II	Trial ongoing	NCT04405349	[106]
ISA101	synthetic long peptides covering the entire HPV16 E6 and E7	Peptide Freunds incomplete adjuvant	Carboplatin/paclitaxel	Advanced, recurrent, or metastatic cervical cancer	I/II	Tumor regression on 43% of patients. HPV T cell responses were mounted after vaccination, and higher responses correlated with longer survival	NCT02128126 and EudraCT 2013-1804-12	[99]
			aPD1	Incurable HPV16-positive cancer (mostly OPSCC)	II	Overall response rate was 33%, and overall survival was 17.5 months. Seems promising compared to aPD1 alone, but a randomized clinical trial to confirm the contribution of ISA101 is needed	NCT02426892	[102]
ADXS11-001	HPV16 E7	Listeria monocytogenes	Cisplatin	Advanced cervical cancer	II (phase III ongoing)	Median overall survival was about 8.5 months with or without cisplatin, with a 12-months overall survival of 30.9–38.9%. Median progression-free survival was 6 months, and the overall response rate was 14.7–17.1%	CTRI/2010/091/ 001232 (phase II trial) NCT02853604 (phase II trial)	[112,113]
TG4001	full length HPV16 E6 and E7	MVA Encoding HPV antigens and IL-2	aPDL-1	Recurrent/metastatic HPV+ cancers (15 anal, 8 OPSCC, 6 cervical, 5 vulvar/vaginal)	Ib/II	In total, 23.5% shows clinical response (1/34 had complete clinical response, 7/34 had partial response), and >50% showed no disease progression at 12 weeks (compared to expected mean PFS of 8 weeks in this population with current treatment). Responders had more CD3 cell infiltration into tumor. PDL1 expression in tumor correlated with better clinical response	NCT03260023	[110,111]
GX-188E	HPV16 and 18 E6 and E7	DNA Encoding HPV antigens and Fms-like tyrosine kinase-3 ligand	aPD1	Recurrent or advanced, inoperable HPV16 or 18+ cervical cancer with progression after standard-of-care therapy	II	Clinical response in 42% of patients at 24 weeks (complete response in 4/36, partial response in 7/36)	NCT03444376	[107]
PDS0101	HPV16 E6 and E7 peptides	Peptides in liposomal nanoparticle	Bintrafusp alfa (targets TGF-b and PDL-1) and NHS-IL12	Advanced HPV− associated malignancies (failed standard of care: chemoradiotherapy and CPI)	II (ongoing)	CPI naïve patients: 83% showed >30% tumor reduction (5/6, compared to 12–24% for standard of care CPI) CPI failed patients: 42% showed clinical response (5/12, compared to 5–12% at current standard of care)	NCT04287868	[104,105]
			aPDL-1	Metastatic HNSCC	II (started mar 21)	Trial ongoing	NCT04260126	
			Chemoradiotherapy	Cervical cancer	II (started oct 20)	Trial ongoing	NCT04580771	
HB-201/HB-202	HPV16 E6/E7 fusion protein	Encoded into LCMV or PICV		HPV16+ head and neck squamous cell carcinoma (HNSCC) and other HPV16+ cancers	I/II	8/18 patients had stable disease 2/18 had partial response	NCT04180215	[114]
SQZ-PBMC-HPV-101	HPV16 E6 and E7 antigens	Antigens are delivered ex vivo to cytosol of patient APCs (using cell squeeze technology)	aPD1, aPDL-1 or aCTLA-4	Incurable HPV16+ cancers	I	4/12 patients achieved stable disease	NCT04084951	[108,109]

## 7. Exhaustion and Implications on Selection of Antigens for Therapeutic Vaccination

The outlined clinical trials have provided promising results but also leave room for improvement. One important question is whether the barrier is technological and could be overcome by new and enhanced vaccine delivery or treatment combinations, or whether the answer lies in a deeper understanding of the intrinsic biological features of HPV infections and HPV+ tumors.

Common for all these therapeutic vaccine candidates is that, despite generally successful mouse data (in a single inbred strain expressing only two polymorphic MHC class I alleles), we are still far from achieving complete lesion regression and clearance of infection, underlining the challenges in the translation from animal model to clinical efficacy. Another similarity is the narrow focus on the HPV oncogenes E6 and E7, and no inclusion of other early HPV genes. In this review, we have presented some arguments for including a wider range of antigenic targets, such as E1 and E2, based on the expression patterns and immunobiology of HPV in HPV+ lesions and cancer. As already mentioned, patient data on correlation between such immune responses and disease outcome would be instructive for future therapeutic vaccine development.

Another reason for looking further than the upregulated viral oncogenes, is exemplified by a study by Stevanovic et al., where HPV+ tumors in a small set of patients had itself induced E6 and E7 specific tumor-infiltrating T cells, but not toward other HPV antigens [118]. Despite these responses, there was no tumor control. Infusion of ex vivo expanded tumor-infiltrating T cells using tumor cells as target, resulted in complete remission of some of the cancers. Interestingly, analysis of the cells used for infusion showed that the expansion had led to increase of non-viral specificities rather than the canonical HPV− antigens. Furthermore, the analysis showed that the initially E6− and E7− reactive T cells all expressed PD1, and they speculate that the lack of tumor control by these T cells could be due to functional exhaustion [118].

This finding by Stevanovic et al. indicates that CPI treatment may convey tumor control by reverting an otherwise exhausted antitumor T cell response. This is supported by another study by Krishna et al., wherein highly expressed HPV+ tumor antigens induced exhausted and dysfunctional T cells [119]. As CPI is being used routinely for advanced HPV+ cancers and in combination with therapeutic vaccination in many clinical trials, it is encouraging that Krishna et al. found that CPI treatment helped overcome the profound dysfunction of the anti-HPV antigen specific T cells [119].The same phenomenon of the implications of exhausted pre-existing T cell responses have been reported for other chronic viral infections, such as Hepatitis C virus (HCV), where a highly immunogenic viral-vectored vaccine induced strong T cell responses in healthy volunteers but impaired and functionally exhausted responses in patients with HCV infection [120]. Importantly, sequencing of HCV in the patients showed that potent T cell responses were only generated when there was a sequence mismatch between autologous virus and vaccine antigen. Another study on Hepatitis B Virus (HBV) showed that high viral titers of HBV at the time of therapeutic vaccination impairs the response as this led to exhaustion of the T cells, but that knockdown of HBV replication before vaccination increased immune responses and led to clearance of infection [121], possibly because the anti-HBV T cells were not terminally exhausted. Either way, the HBV study indicates that high antigen loads lead to exhaustion, which may be the case for HPV as well. These findings underscore the danger of ignoring exhaustion in advanced diseases, even with immunogenic vaccine regimens, and highlight the need for CPI. It may also help explain the lack of clinical response of the HB-200 therapy, Despite the impressive T cell responses.

As E1 is not naturally immunogenic in at least cervical cancers, but is expressed in the tumor, and has been suggested to play a role in carcinogenesis [46]; this could stipulate a potential non-exhausted target. Additionally, the size of the antigens argues for the benefits of including E1 and E2, as previously mentioned, allowing for many more potential epitopes, thus catering for the large variation of HLA-alleles as present in humans.

## 8. Conclusive Remarks

The need for therapies against HPV+ malignancies is clear, and the transition toward HPV− detection in screening programs provides a window of opportunity for HPV− targeted therapeutic approaches. Therapeutic vaccines have shown promising results, both against pre-cancerous lesions and in HPV+ cancers, often in combination with other treatments such a chemoradiotherapy or CPI. Especially, combination treatment with vaccines and CPI may be promising, as this may circumvent the potential exhaustion of HPV− specific cytotoxic T cells induced by the chronic HPV infection.

However, there is still some way to go in the development of highly effective therapeutic vaccines against HPV and HPV-associated cancers. This journey will be aided by better understanding of the underlying biology of the HPV-associated malignancies and the interplay with our immune system. Here, we proposed that a broader antigenic focus, including more HPV proteins than E6 and E7 in therapeutic vaccines, which may be an important step toward better therapeutic vaccines, and we suggested that host genetics at the HLA level should be reported when publishing therapeutic HPV vaccine studies.

To the best of our knowledge, the only recently tested therapeutic encompassing other antigens than E6 and E7 is the previously mentioned ChAdOx- and MVA-vectored vaccine (VTP-200), encoding conserved elements of E1, E2, E4, E5, E6 and E7 proteins representing HPV genotypes 16, 18, 31, 52 and 58. This is currently being tested in a phase I/II placebo-controlled trial against low grade cervical lesions. Results from this trial will be instructive for developing the paradigm of targeting HPV antigens beyond E6 and E7, although it is uncertain how detrimental the attempted breadth of targeting will be for efficacy against individual types. We have found no published clinical trials on cancer patients with therapeutic vaccines targeting other HPV proteins than E1 and E2.

## References

[1] Chaturvedi A.K., Engels E.A., Pfeiffer R.M., Hernandez B.Y., Xiao W., Kim E., Jiang B., Goodman M.T., Sibug-Saber M., Cozen W. (2011). Human papillomavirus and rising oropharyngeal cancer incidence in the United States. J. Clin. Oncol..

[2] Gillison M.L., Koch W.M., Capone R.B., Spafford M., Westra W.H., Wu L., Zahurak M.L., Daniel R.W., Viglione M., Symer D.E. (2000). Evidence for a causal association between human papillomavirus and a subset of head and neck cancers. J. Natl. Cancer Inst..

[3] De Martel C., Georges D., Bray F., Ferlay J., Clifford G.M. (2020). Global burden of cancer attributable to infections in 2018: A worldwide incidence analysis. Lancet Glob. Health.

[4] Bray F., Ferlay J., Soerjomataram I., Siegel R.L., Torre L.A., Jemal A. (2018). Global cancer statistics 2018: GLOBOCAN estimates of incidence and mortality worldwide for 36 cancers in 185 countries. CA Cancer J. Clin..

[5] Dillon S., Sasagawa T., Crawford A., Prestidge J., Inder M.K., Jerram J., Mercer A., Hibma M. (2007). Resolution of cervical dysplasia is associated with T-cell proliferative responses to human papillomavirus type 16 E2. J. Gen. Virol..

[6] Bruni L., Saura-Lázaro A., Montoliu A., Brotons M., Alemany L., Diallo M.S., Afsar O.Z., LaMontagne D.S., Mosina L., Contreras M. (2021). HPV vaccination introduction worldwide and WHO and UNICEF estimates of national HPV immunization coverage 2010–2019. Prev. Med..

[7] World Health Organization (WHO) (2020). Accelerating the Elimination of Cervical Cancer as a Global Public Health Problem.

[8] Kyndi M., Frederiksen K., Kjær S.K., Kjaer S. (2006). Cervical cancer incidence in Denmark over six decades (1943–2002). Acta Obstet. Gynecol. Scand..

[9] Lynge E., Rygaard C., Baillet M.V.-P., Dugué P.-A., Sander B.B., Bonde J., Rebolj M. (2014). Cervical cancer screening at crossroads. APMIS.

[10] Aklimunnessa K., Mori M., Khan M.M.H., Sakauchi F., Kubo T., Fujino Y., Suzuki S., Tokudome S., Tamakoshi A. (2006). Effectiveness of cervical cancer screening over cervical cancer mortality among japanese women. Jpn. J. Clin. Oncol..

[11] Garcia-Hernandez E., Gonzalez-Sanchez J.L., Andrade-Manzano A., Contreras M.L., Padilla S., Guzman C.C., Jimenez R., Reyes L., Morosoli G., Verde M.L. (2006). Regression of papilloma high-grade lesions (CIN 2 and CIN 3) is stimulated by therapeutic vac-cination with MVA E2 recombinant vaccine. Cancer Gene Ther..

[12] Rodriguez-Freixinos V., Mackay H.J. (2015). Breaking down the evidence for bevacizumab in advanced cervical cancer: Past, present and future. Gynecol. Oncol. Res. Pr..

[13] Landoni F., Maneo A., Colombo A., Placa F., Milani R., Perego P., Favini G., Ferri L., Mangioni C. (1997). Randomised study of radical surgery versus radiotherapy for stage Ib-IIa cervical cancer. Lancet.

[14] Zamani M., Grønhøj C., Jensen D.H., Carlander A.F., Agander T., Kiss K., Olsen C., Baandrup L., Nielsen F.C., Andersen E. (2020). The current epidemic of HPV-associated oropharyngeal cancer: An 18-year Danish population-based study with 2,169 patients. Eur. J. Cancer.

[15] Steinau M., Saraiya M., Goodman M.T., Peters E.S., Watson M., Cleveland J.L., Lynch C.F., Wilkinson E.J., Hernandez B.Y., Copeland G. (2014). Human papillomavirus prevalence in oropharyngeal cancer before vaccine introduction, United States. Emerg. Infect. Dis..

[16] Kreimer A.R., Shiels M.S., Fakhry C., Johansson M., Pawlita M., Brennan P., Hildesheim A., Waterboer T. (2018). Screening for human papillomavirus-driven oropharyngeal cancer: Considerations for feasibility and strategies for research. Cancer.

[17] D’Souza G., Clemens G., Troy T., Castillo R.G., Struijk L., Waterboer T., Bender N., Pierorazio P.M., Best S.R., Strickler H.D. (2019). Evaluating the utility and prevalence of HPV biomarkers in oral rinses and serology for HPV-related oropharyngeal cancer. Cancer Prev. Res..

[18] Chera B.S., Kumar S., Shen C., Amdur R., Dagan R., Green R., Goldman E., Weiss J., Grilley-Olson J., Patel S. (2020). Plasma circulating tumor HPV DNA for the surveillance of cancer recurrence in HPV-associated oro-pharyngeal cancer. J. Clin. Oncol..

[19] Ang K.K., Harris J., Wheeler R., Weber R., Rosenthal D.I., Nguyen-Tân P.F., Westra W.H., Chung C.H., Jordan R.C., Lu C. (2010). Human papillomavirus and survival of patients with oropharyngeal cancer. N. Engl. J. Med..

[20] Wuerdemann N., Gültekin S.E., Pütz K., Wittekindt C., Huebbers C.U., Sharma S.J., Eckel H., Schubotz A.B., Gattenlöhner S., Büttner R. (2020). PD-L1 expression and a high tumor infiltrate of CD8+ lymphocytes predict outcome in patients with oropharyngeal squamous cells carcinoma. Int. J. Mol. Sci..

[21] Kjems J., Gothelf A.B., Håkansson K., Specht L., Kristensen C.A., Friborg J. (2016). Elective nodal irradiation and patterns of failure in head and neck cancer after primary radiation therapy. Int. J. Radiat. Oncol..

[22] Bauml J., Seiwert T.Y., Pfister D.G., Worden F., Liu S.V., Gilbert J., Saba N.F., Weiss J., Wirth L., Sukari A. (2017). Pembrolizumab for platinum- and cetuximab-refractory head and neck cancer: Results from a single-arm, phase II study. J. Clin. Oncol..

[23] Ferris R.L., Blumenschein G., Fayette J., Guigay J., Colevas A.D., Licitra L., Harrington K., Kasper S., Vokes E.E., Even C. (2016). Nivolumab for recurrent squamous-cell carcinoma of the head and neck. N. Engl. J. Med..

[24] Xu Y., Zhu G., Maroun C.A., Wu I.X.Y., Huang D., Seiwert T.Y., Liu Y., Mandal R., Zhang X. (2021). Programmed death-1/programmed death-ligand 1-Axis blockade in recurrent or metastatic head and neck squamous cell carcinoma stratified by human papillomavirus status: A systematic review and meta-analysis. Front. Immunol..

[25] Waggoner S.E. (2003). Cervical cancer. Lancet.

[26] Chung H., Ros W., Delord J.-P., Perets R., Italiano A., Shapira-Frommer R., Manzuk L., Piha-Paul S., Xu L., Zeigenfuss S. (2019). Efficacy and safety of pembrolizumab in previously treated advanced cervical cancer: Results from the phase II KEYNOTE-158 study. J. Clin. Oncol..

[27] Spanos W.C., Nowicki P., Lee D.W., Hoover A., Hostager B., Gupta A., Anderson M.E., Lee J.H. (2009). Immune response during therapy with cisplatin or radiation for human papillomavirus–related head and neck cancer. Arch. Otolaryngol.-Head Neck Surg..

[28] Cosper P.F., McNair C., González I., Wong N., Knudsen K.E., Chen J.J., Markovina S., Schwarz J.K., Grigsby P.W., Wang X. (2020). Decreased local immune response and retained HPV gene expression during chemoradiotherapy are associated with treatment resistance and death from cervical cancer. Int. J. Cancer.

[29] Hancock G., Hellner K., Dorrell L. (2018). Therapeutic HPV vaccines. Best Pr. Res. Clin. Obstet. Gynaecol..

[30] Wang R., Pan W., Jin L., Huang W., Li Y., Wu D., Gao C., Ma D., Liao S. (2020). Human papillomavirus vaccine against cervical cancer: Opportunity and challenge. Cancer Lett..

[31] Schiller J.T., Müller M. (2015). Next generation prophylactic human papillomavirus vaccines. Lancet Oncol..

[32] Narisawa-Saito M., Kiyono T. (2007). Basic mechanisms of high-risk human papillomavirus-induced carcinogenesis: Roles of E6 and E7 proteins. Cancer Sci..

[33] Khwaja S.S., Baker C., Haynes W., Spencer C.R., Gay H., Thorstad W., Adkins D.R., Nussenbaum B., Chernock R.D., Lewis J.S. (2016). High E6 gene expression predicts for distant metastasis and poor survival in patients with HPV-positive oropharyngeal squamous cell carcinoma. Int. J. Radiat. Oncol..

[34] Dong G., Broker T.R., Chow L.T. (1994). Human papillomavirus type 11 E2 proteins repress the homologous E6 promoter by interfering with the binding of host transcription factors to adjacent elements. J. Virol..

[35] Baker C.C., Phelps W.C., Lindgren V.A., Braun M.J., Gonda M.A., Howley P.M. (1987). Structural and transcriptional analysis of human papillomavirus type 16 sequences in cervical carcinoma cell lines. J. Virol..

[36] Xue Y., Bellanger S., Zhang W., Lim D., Low J., Lunny D., Thierry F. (2010). HPV16 E2 is an immediate early marker of viral infection, preceding E7 expression in precursor structures of cervical carcinoma. Cancer Res..

[37] Waxman A.G., Chelmow D., Darragh T.M., Lawson H., Moscicki A.-B. (2012). Revised terminology for cervical histopathology and its implications for management of high-grade squamous intraepithelial lesions of the cervix. Obstet. Gynecol..

[38] Arias-Pulido H., Peyton C.L., Joste N.E., Vargas H., Wheeler C.M. (2006). Human papillomavirus type 16 integration in cervical carcinoma in situ and in invasive cervical cancer. J. Clin. Microbiol..

[39] Nkili-Meyong A.A., Moussavou-Boundzanga P., Labouba I., Koumakpayi I.H., Jeannot E., Descorps-Declère S., Sastre-Garau X., Leroy E.M., Belembaogo E., Berthet N. (2019). Genome-wide profiling of human papillomavirus DNA integration in liquid-based cytology speci-mens from a Gabonese female population using HPV capture technology. Sci. Rep..

[40] Balaji H., Demers I., Wuerdemann N., Schrijnder J., Kremer B., Klussmann J.P., Huebbers C.U., Speel E.-J.M. (2021). Causes and consequences of HPV integration in head and neck squamous cell carcinomas: State of the art. Cancers.

[41] Pett M., Coleman N. (2007). Integration of high-risk human papillomavirus: A key event in cervical carcinogenesis?. J. Pathol..

[42] Morgan I.M., Dinardo L.J., Windle B. (2017). Integration of human papillomavirus genomes in head and neck cancer: Is it time to consider a paradigm shift?. Viruses.

[43] Vinokurova S., Wentzensen N., Kraus I., Klaes R., Driesch C., Melsheimer P., Kisseljov F., Dürst M., Schneider A., Doeberitz M.V.K. (2008). Type-dependent integration frequency of human papillomavirus genomes in cervical lesions. Cancer Res..

[44] Anayannis N.V., Schlecht N.F., Ben-Dayan M., Smith R.V., Belbin T.J., Ow T.J., Blakaj D.M., Burk R.D., Leonard S.M., Woodman C.B. (2018). Association of an intact E2 gene with higher HPV viral load, higher viral oncogene expression, and improved clinical outcome in HPV16 positive head and neck squamous cell carcinoma. PLoS ONE.

[45] Bergvall M., Melendy T., Archambault J. (2013). The E1 proteins. Virology.

[46] Baedyananda F., Chaiwongkot A., Bhattarakosol P. (2018). Elevated HPV16 E1 expression is associated with cervical cancer progression. Intervirology.

[47] Ewaisha R., Panicker G., Maranian P., Unger E.R., Anderson K.S. (2017). Serum immune profiling for early detection of cervical disease. Theranostics.

[48] Malcles M.-H., Cueille N., Mechali F., Coux O., Bonne-Andrea C. (2002). Regulation of bovine papillomavirus replicative helicase E1 by the ubiquitin-proteasome pathway. J. Virol..

[49] Mechali F., Hsu C.-Y., Castro A., Lorca T., Bonne-Andrea C. (2004). Bovine papillomavirus replicative helicase E1 is a target of the ubiquitin ligase APC. J. Virol..

[50] Leachman S.A., Shylankevich M., Slade M.D., Levine D., Sundaram R.K., Xiao W., Bryan M., Zelterman D., Tiegelaar R.E., Brandsma J.L. (2002). Ubiquitin-fused and/or multiple early genes from cottontail rabbit papillomavirus as DNA vaccines. J. Virol..

[51] Esposito I., Cicconi P., Capone S., Brown A., Esposito M.L., Mori F., Vassilev V., Siani L., Ghaffari E., Gardiner C. (2019). GS-05-MHC-II invariant chain adjuvanted chimpanzee adenoviral and MVA hepatitis C vaccines elicit un-precedented levels of anti-viral T-cell immune responses in humans. J. Hepatology.

[52] Ma M., Feng Y., Fan P., Yao X., Peng Y., Dong T., Wang R. (2018). Human papilloma virus E1-specific T cell immune response is associated with the prognosis of cervical cancer patients with squamous cell carcinoma. Infect. Agents Cancer.

[53] Nakagawa M., Stites D.P., Patel S., Farhat S., Scott M., Hills N.K., Palefsky J.M., Moscicki A. (2000). Persistence of human papillomavirus type 16 infection is associated with lack of cytotoxic t lymphocyte response to the E6 antigens. J. Infect. Dis..

[54] Piersma S., Jordanova E.S., Van Poelgeest M.I., Kwappenberg K.M., Van Der Hulst J.M., Drijfhout J.W., Melief C.J., Kenter G., Fleuren G.J., Offringa R. (2007). High number of intraepithelial CD8+ tumor-infiltrating lymphocytes is associated with the absence of lymph node metastases in patients with large early-stage cervical cancer. Cancer Res..

[55] Masterson L., Lechner M., Loewenbein S., Mohammed H., Davies-Husband C., Fenton T., Sudhoff H., Jani P., Goon P., Sterling J. (2016). CD8 + T cell response to human papillomavirus 16 E7 is able to predict survival outcome in oropharyngeal cancer. Eur. J. Cancer.

[56] Woo Y.L., Hende M.V.D., Sterling J.C., Coleman N., Crawford R.A.F., Kwappenberg K.M.C., Stanley M.A., Van Der Burg S.H. (2009). A prospective study on the natural course of low-grade squamous intraepithelial lesions and the presence of HPV16 E2-, E6- and E7-specific T-cell responses. Int. J. Cancer.

[57] Ewaisha R., Meshay I., Resnik J., Katchman B.A., Anderson K.S. (2016). Inside front cover: Programmable protein arrays for immunoprofiling HPV-associated cancers. Proteomics.

[58] Anderson K.S., Dahlstrom K., Cheng J.N., Alam R., Li G., Wei Q., Gross N.D., Chowell D., Posner M., Sturgis E.M. (2015). HPV16 antibodies as risk factors for oropharyngeal cancer and their association with tumor HPV and smoking status. Oral Oncol..

[59] Dahlstrom K., Anderson K.S., Cheng J.N., Chowell D., Li G., Posner M.R., Sturgis E.M. (2015). HPV serum antibodies as predictors of survival and disease progression in patients with HPV-positive squamous cell carcinoma of the oropharynx. Clin. Cancer Res..

[60] Bhatt K.H., Neller M.A., Srihari S., Crooks P., Lekieffre L., Aftab B.T., Liu H., Smith C., Kenny L., Porceddu S. (2020). Profiling HPV-16–specific T cell responses reveals broad antigen reactivities in oropharyngeal cancer patients. J. Exp. Med..

[61] Yajid A.I., Zakariah M.A., Zin A.A.M., Othman N.H. (2017). Potential role of E4 protein in human papillomavirus screening: A review. Asian Pac. J. Cancer Prev..

[62] Campo M., Graham S., Cortese M., Ashrafi G., Araibi E., Dornan E., Miners K., Nunes C., Man S. (2010). HPV-16 E5 down-regulates expression of surface HLA class I and reduces recognition by CD8 T cells. Virology.

[63] Hausen H.Z. (2002). Papillomaviruses and cancer: From basic studies to clinical application. Nat. Rev. Cancer.

[64] Lorenzon L., Mazzetta F., Venuti A., Frega A., Torrisi M.R., French D. (2011). In vivo HPV 16 E5 mRNA: Expression pattern in patients with squamous intra-epithelial lesions of the cervix. J. Clin. Virol..

[65] Um S.H., Mundi N., Yoo J., A Palma D., Fung K., MacNeil D., Wehrli B., Mymryk J.S., Barrett J.W., Nichols A.C. (2014). Variable expression of the forgotten oncogene E5 in HPV-positive oropharyngeal cancer. J. Clin. Virol..

[66] Todd R.W., Roberts S., Mann C.H., Luesley D.M., Gallimore P.H., Steele J.C. (2004). Human papillomavirus (HPV) type 16-specific CD8+ T cell responses in women with high grade vulvar intraepithelial neoplasia. Int. J. Cancer.

[67] Ramqvist T., Mints M., Tertipis N., Näsman A., Romanitan M., Dalianis T. (2015). Studies on human papillomavirus (HPV) 16 E2, E5 and E7 mRNA in HPV-positive tonsillar and base of tongue cancer in relation to clinical outcome and immunological parameters. Oral Oncol..

[68] Eberhardt C.S., Kissick H.T., Patel M.R., Cardenas M.A., Prokhnevska N., Obeng R.C., Nasti T.H., Griffith C.C., Im S.J., Wang X. (2021). Functional HPV-specific PD-1+ stem-like CD8 T cells in head and neck cancer. Nat. Cell Biol..

[69] Rijkaart D.C., Berkhof J., Rozendaal L., van Kemenade F.J., Bulkmans N.W., Heideman D.A., Kenter G., Cuzick J., Snijders P.J., Meijer C.J. (2012). Human papillomavirus testing for the detection of high-grade cervical intraepithelial neoplasia and cancer: Final results of the POBASCAM randomised controlled trial. Lancet Oncol..

[70] Nkwabong E., Badjan I.L.B., Sando Z. (2018). Pap smear accuracy for the diagnosis of cervical precancerous lesions. Trop. Dr..

[71] Perkins R.B., Guido R.S., Castle P.E., Chelmow D., Einstein M.H., Garcia F., Huh W.K., Kim J.J., Moscicki A.-B., Nayar R. (2020). 2019 ASCCP Risk-based management consensus guidelines for abnormal cervical cancer screening tests and cancer precursors. J. Low. Genit. Tract Dis..

[72] Fontham E.T.H., Wolf A.M.D., Church T.R., Etzioni R., Flowers C.R., Herzig A., Guerra C.E., Oeffinger K.C., Shih Y.T., Walter L.C. (2020). Cervical cancer screening for individuals at average risk: 2020 guideline update from the American Cancer Society. CA A Cancer J. Clin..

[73] Australian Government-National Cervical Screening Program. https://www.health.gov.au/initiatives-and-programs/national-cervical-screening-program/about-the-national-cervical-screening-program#the-new-cervical-screening-test-is-more-effective.

[74] Kreftregisteret HPV I Primærscreening. https://www.kreftregisteret.no/screening/livmorhalsprogrammet/Helsepersonell/screeningstrategi-og-nasjonale-retningslinjer/HPV-i-primarscreening/.

[75] New Zealand Government-Ministry of Health (2017). Primary HPV Screening. https://www.nsu.govt.nz/health-professionals/national-cervical-screening-programme/hpv-primary-screening.

[76] UK Government (2019). The UK National Screening Committee Recommendation on Cervical Cancer Screening in Women. https://view-health-screening-recommendations.service.gov.uk/cervical-cancer/.

[77] (2015). Screening för Livmoderhalscancer Rekommendation Och Bedömningsunderlag. https://www.socialstyrelsen.se/globalassets/sharepoint-dokument/artikelkatalog/nationella-screeningprogram/2015-6-39.pdf.

[78] (2018). Statens Serum Institut-Screening for Livmoderhals-kræft-Anbefalinger. https://www.sst.dk/da/Udgivelser/2018/Screening-for-livmoderhalskraeft.

[79] Schmitt M., Pawlita M. (2011). The HPV transcriptome in HPV16 positive cell lines. Mol. Cell. Probes.

[80] Dorta-Estremera S., Chin R., Sierra G., Nicholas C., Yanamandra A.V., Nookala S.M., Yang G., Singh S., Curran M., Sastry K.J. (2018). Mucosal HPV E6/E7 peptide vaccination in combination with immune checkpoint modulation induces regression of HPV+ oral cancers. Cancer Res..

[81] Çuburu N., Schiller J.T. (2016). Moving forward with human papillomavirus immunotherapies. Hum. Vaccines Immunother..

[82] Van Poelgeest M.I., Welters M.J., Vermeij R., Stynenbosch L.F., Loof N.M., Berends-van der Meer D.M., Löwik M.J., Hamming I.L., van Esch E.M., Hellebrekers B.W. (2016). Vaccination against oncoproteins of HPV16 for noninvasive vulvar/vaginal lesions: Lesion clearance is related to the strength of the T-cell response. Clin. Cancer Res..

[83] Kenter G.G., Welters M.J.P., Valentijn A.R.P.M., Lowik M.J.G., Der Meer D.M.A.B.-V., Vloon A.P.G., Essahsah F., Fathers L.M., Offringa R., Drijfhout J.W. (2009). Vaccination against HPV-16 oncoproteins for vulvar intraepithelial neoplasia. N. Engl. J. Med..

[84] Preville X., Ladant D., Timmerman B., Leclerc C. (2005). Eradication of established tumors by vaccination with recombinant Bordetella pertussis adenylate cyclase carrying the human papillomavirus 16 E7 oncoprotein. Cancer Res..

[85] Van Damme P., Bouillette-Marussig M., Hens A., De Coster I., Depuydt C., Goubier A., Van Tendeloo V., Cools N., Goossens H., Hercend T. (2016). GTL001, A Therapeutic vaccine for women infected with human papillomavirus 16 or 18 and normal cervical cytology: Results of a phase I clinical trial. Clin. Cancer Res..

[86] Genticel (2016). Press Release: Genticel Reports Final Results of GTL001 Phase 2 Trial in HPV16/18-Infected Women. https://www.genkyotex.com/images/PDF/GB/1_Press_Releases/2016/ACTUS-0-46696-161213_PR_final_ph2_results_GTL001_VDEF.pdf.

[87] Trimble C.L., Morrow M.P., Kraynyak K.A., Shen X., Dallas M., Yan J., Edwards L., Parker R.L., Denny L., Giffear M. (2015). Safety, efficacy, and immunogenicity of VGX-3100, a therapeutic synthetic DNA vaccine targeting human papillomavirus 16 and 18 E6 and E7 proteins for cervical intraepithelial neoplasia 2/3: A randomised, double-blind, place-bo-controlled phase 2b trial. Lancet.

[88] Bhuyan P.K., Dallas M., Kraynyak K., Herring T., Morrow M., Boyer J., Duff S., Kim J., Weiner D.B. (2021). Durability of response to VGX-3100 treatment of HPV16/18 positive cervical HSIL. Hum. Vaccines Immunother..

[89] Choi Y.J., Hur S.Y., Kim T.-J., Hong S.R., Lee J.K., Cho C.-H., Park K.S., Woo J.W., Sung Y.C., Suh Y.S. (2019). A Phase II, prospective, randomized, multicenter, open-label study of gx-188e, an hpv dna vaccine, in patients with cervical intraepithelial neoplasia 3. Clin. Cancer Res..

[90] Hillemanns P., Petry K.U., Woelber L., Böhmer G., Stubsrud E., Skjørestad I., Schjetne K., Fredriksen A., Axelsen M. Abstract CT209: Safety, efficacy and immunogenicity of VB10.16, a therapeutic DNA vaccine targeting human papillomavirus (HPV) 16 E6 and E7 proteins for high grade cervical intraepithelial neoplasia (CIN 2/3): 6-month data from an exploratory open-label phase I/2a trial. Proceedings of the Clinical Trials; American Association for Cancer Research (AACR).

[91] Vaccibody (2019). Press Release: Positive 12-Month Results from Phase IIA Clinical Study in High Grade Cer-Vical Dysplasia Provides Proof-Of-Concept for Vaccibody’s Immunotherapy Platform and Lead Candidate VB1O.16. https://www.vaccibody.com/positive-12-month-results-from-phase-iia-clinical-study-in-high-grade-cervical-dysplasia-provides-proof-of-concept-for-vaccibodys-immunotherapy-platform-and-lead-candidate-vb1o-16/.

[92] Harper D.M., Nieminen P., Donders G., Einstein M.H., Garcia F., Huh W.K., Stoler M.H., Glavini K., Attley G., Limacher J.-M. (2019). The efficacy and safety of Tipapkinogen Sovacivec therapeutic HPV vaccine in cervical intraepithelial neoplasia grades 2 and 3: Randomized controlled phase II trial with 2.5 years of follow-up. Gynecol. Oncol..

[93] Maldonado L., Teague J.E., Morrow M.P., Jotova I., Wu T.C., Wang C., Desmarais C., Boyer J.D., Tycko B., Robins H.S. (2014). Intramuscular therapeutic vaccination targeting HPV16 Induces T cell responses that localize in mucosal lesions. Sci. Transl. Med..

[94] Hancock G., Blight J., Lopez-Camacho C., Kopycinski J., Pocock M., Byrne W., Price M.J., Kemlo P., Evans R.I., Bloss A. (2019). A multi-genotype therapeutic human papillomavirus vaccine elicits potent T cell responses to conserved regions of early proteins. Sci. Rep..

[95] ClinicalTrialsRegister.eu A Phase 1b/2 Randomised, Placebo-controlled, Dose-ranging Study to Evaluate Safety, Tolerability and Immunogenicity of a Chimpanzee Adenovirus (ChAdOx1)-vectored Multigenotype High Risk Human Papillomavirus (hrHPV) Vaccine and Modified Vaccinia Ankara (MV. EudraCT 2019-001890-98, HPV001)..

[96] Ragonnaud E., Andersson A.-M.C., Mariya S., Pedersen A.G., Burk R.D., Folgori A., Colloca S., Cortese R., Nicosia A., Pamungkas J. (2017). Therapeutic vaccine against primate papillomavirus infections of the cervix. J. Immunother..

[97] Ragonnaud E., Pedersen A.G., Holst P.J. (2017). Breadth of T cell responses after immunization with adenovirus vectors en-coding ancestral antigens or polyvalent papillomavirus antigens. scand. Scand. J. Immunol..

[98] Rosales R., López-Contreras M., Rosales C., Magallanes-Molina J.-R., Gonzalez-Vergara R., Arroyo-Cazarez J.M., Ricardez-Arenas A., Del Follo-Valencia A., Padilla-Arriaga S., Guerrero M.V. (2014). Regression of Human Papillomavirus Intraepithelial Lesions Is Induced by MVA E2 Therapeutic Vaccine. Hum. Gene Ther..

[99] Melief C.J.M., Welters M.J.P., Vergote I., Kroep J.R., Kenter G.G., Ottevanger P.B., Tjalma W.A.A., Denys H., van Poelgeest M.I.E., Nijman H.W. (2020). Strong vaccine responses during chemotherapy are associated with prolonged cancer survival. Sci. Transl. Med..

[100] Lorusso D., Petrelli F., Coinu A., Raspagliesi F., Barni S. (2014). A systematic review comparing cisplatin and carboplatin plus paclitaxel-based chemotherapy for recurrent or metastatic cervical cancer. Gynecol. Oncol..

[101] Hladíková K., Partlová S., Koucký V., Bouček J., Fonteneau J.-F., Zábrodský M., Tachezy R., Grega M., Špíšek R., Fialová A. (2018). Dysfunction of HPV16-specific CD8+ T cells derived from oropharyngeal tumors is related to the expression of Tim-3 but not PD-1. Oral Oncol..

[102] Massarelli E., William W., Johnson F., Kies M., Ferrarotto R., Guo M., Feng L., Lee J.J., Tran H., Kim Y.U. (2019). Combining immune checkpoint blockade and tumor-specific vaccine for patients with incurable human papillomavirus 16–related cancer. JAMA Oncol..

[103] Mehra R., Seiwert T.Y., Gupta S., Weiss J., Gluck I., Eder J.P., Burtness B., Tahara M., Keam B., Kang H. (2018). Efficacy and safety of pembrolizumab in recurrent/metastatic head and neck squamous cell carcinoma: Pooled analyses after long-term follow-up in KEYNOTE-012. Br. J. Cancer.

[104] Strauss J., Floudas C.S., Sater H.A., Manu M., Lamping E., Francis D.C., Cordes L.M., Marte J., Donahue R.N., Jochems C. (2021). Phase II evaluation of the triple combination of PDS0101, M9241, and bintrafusp alfa in patients with HPV 16 positive malignancies. J. Clin. Oncol..

[105] PDS Biotechnology Corporation (2021). PDS Biotech Announces Release of Interim Data for PDS0101 in NCI-Led Phase 2 Clinical Study in Oral Presentation at ASCO 2021 Annual Meeting. GLobe Newswire..

[106] Hillemanns P., Baurain J.-F., Blecharz P., Lindemann K., Nicolaisen B., Schetne K., Fredriksen A., Torhaug S. (2020). 881TiP A multi-centre, open-label phase II trial of the combination of VB10.16 and atezolizumab in patients with advanced or recurrent, non-resectable HPV16 positive cervical cancer. Ann. Oncol..

[107] Youn J.W., Hur S.-Y., Woo J.W., Kim Y.-M., Lim M.C., Park S.Y., Seo S.S., No J.H., Kim B.-G., Lee J.-K. (2020). Pembrolizumab plus GX-188E therapeutic DNA vaccine in patients with HPV-16-positive or HPV-18-positive advanced cervical cancer: Interim results of a single-arm, phase 2 trial. Lancet Oncol..

[108] (2020). POSTER: SQZ-PBMC-HPV, an Innovative, Autologous Therapeutic HPV-16+ Cancer Vaccine Engineered by Microfluidic Cell Squeezing to Elicit Robust CD8+ T Cell Responses. IPVC. https://investors.sqzbiotech.com/files/doc_downloads/poster_publications/2020/11/IPVC-2020-Poster-O.-Rosen.pdf.

[109] Jimeno A., Baranda J.C., Mita M.M., Gordon M.S., Taylor M.H., Iams W.T., Janku F., Matulonis U.A., Bernstein H., Loughhead S. (2021). Initial results of a first-in-human, dose escalation study of a cell-based vaccine in HLA A*02+ patients (pts) with recurrent, locally advanced or metastatic HPV16+ solid tumors: SQZ-PBMC-HPV-101. J. Clin. Oncol..

[110] Tourneau C.L. ESMO Abstract: TG4001 Therapeutic Vaccination Plus Avelumab-Mediated PD-L1 Blockade Improves Tumour Microenvironment in HPV-positive malignancies. ESMO Immu-No-Oncology Virtual Congress 2020. https://www.esmo.org/oncology-news/tg4001-therapeutic-vaccination-plus-avelumab-mediated-pd-l1-blockade-improves-tumour-microenvironment-in-hpv-positive-malignancies.

[111] Transgene: Acceptance of Late Breaking Abstract at Upcoming SITC 2020 Conference, on the Detailed Results from Clinical Study of TG4001 in Combination with Avelumab in Advanced HPV-positive Cancers. Business wire 2020. https://www.businesswire.com/news/home/20201018005012/en/Transgene-Acceptance-of-Late-Breaking-Abstract-at-Upcoming-SITC-2020-Conference-on-The-Detailed-Results-From-Clinical-Study-of-TG4001-in-Combination-With-Avelumab-in-Advanced-HPV-positive-Cancers.

[112] Galicia-Carmona T., Arango-Bravo E., a Serrano-Olvera J., La Torre C.F.-D., Cruz-Esquivel I., Villalobos-Valencia R., Morán-Mendoza A., Castro-Eguiluz D., Cetina-Pérez L. (2021). ADXS11-001 LM-LLO as specific immunotherapy in cervical cancer. Hum. Vaccines Immunother..

[113] Basu P., Mehta A., Jain M., Gupta S., Nagarkar R.V., John S., Petit R. (2018). A randomized phase 2 study of ADXS11-001 listeria monocytogenes–listeriolysin O immunotherapy with or without cisplatin in treatment of advanced cervical cancer. Int. J. Gynecol. Cancer.

[114] Ho A.L., Posner M.R., Niu J., Fu S., Leidner R.S., Pearson A.T., Chung K.Y., Richardson D.L., Wang D., Pimentel A. (2021). First report of the safety/tolerability and preliminary antitumor activity of HB-201 and HB-202, an arenavirus-based cancer immunotherapy, in patients with HPV16+ cancers. J. Clin. Oncol..

[115] Verma V., Shrimali R.K., Ahmad S., Dai W., Wang H., Lu S., Nandre R., Gaur P., Lopez J., Sade-Feldman M. (2019). PD-1 blockade in subprimed CD8 cells induces dysfunctional PD-1+CD38hi cells and anti-PD-1 resistance. Nat. Immunol..

[116] Kotturi M.F., Peters B., Buendia-Laysa F., Sidney J., Oseroff C., Botten J., Grey H., Buchmeier M.J., Sette A. (2007). The CD8 + T-cell response to lymphocytic choriomeningitis virus involves the L antigen: Uncovering new tricks for an old virus. J. Virol..

[117] Holst P.J., Sørensen M.R., Jensen C.M.M., Orskov C., Thomsen A.R., Christensen J. (2008). MHC class II-associated invariant chain linkage of antigen dramatically improves cell-mediated immunity induced by adenovirus vaccines. J. Immunol..

[118] Stevanović S., Pasetto A., Helman S.R., Gartner J.J., Prickett T.D., Howie B., Robins H.S., Robbins P.F., Klebanoff C.A., Rosenberg S.A. (2017). Landscape of immunogenic tumor antigens in successful immunotherapy of virally induced epithelial cancer. Science.

[119] Krishna S., Ulrich P., Wilson E., Parikh F., Narang P., Yang S., Read A.K., Kim-Schulze S., Park J.G., Posner M. (2018). Human papilloma virus specific immunogenicity and dysfunction of CD8+ T cells in head and neck cancer. Cancer Res..

[120] Swadling L., Halliday J., Kelly C., Brown A., Capone S., Ansari M.A., Bonsall D., Richardson R., Hartnell F., Collier J. (2016). Highly-immunogenic virally-vectored T-cell vaccines cannot overcome subversion of the T-cell response by HCV during chronic infection. Vaccines.

[121] Michler T., Kosinska A.D., Festag J., Bunse T., Su J., Ringelhan M., Imhof H., Grimm D., Steiger K., Mogler C. (2020). Knockdown of virus antigen expression increases therapeutic vaccine efficacy in high-titer hepatitis B virus carrier mice. Gastroenterology.

